# Perceptions of being a registered nurse (PRN): development and validation of a survey tool

**DOI:** 10.1186/s12912-023-01324-7

**Published:** 2023-05-10

**Authors:** Louise Allen, Simon Cooper, Karen Missen

**Affiliations:** 1 Institute of Health and Wellbeing, Federation University, Room 2W-144, Gippsland Campus, Churchill, VIC Australia; 2 Healthcare Research, Institute of Health and Wellbeing, Federation University, Berwick Campus, Berwick, VIC Australia; 3 Institute of Health and Wellbeing, Federation University, Gippsland Campus, Churchill, VIC Australia

**Keywords:** Perceptions, Nursing students, Nursing, Survey tool, Education

## Abstract

**Background:**

Nursing students enter nursing programs with idealistic perceptions of what it is to be a nurse. Upon graduation, many find these perceptions mismatched with the actual nurse’s role. This can lead to discontentment in their chosen career. These issues highlight the importance of nursing students developing an understanding of the nurse’s role during their undergraduate nursing education. One way to accomplish this is to assess perceptions and address them accordingly during the nursing program. Survey tools assessing perceptions of nursing exist but lack contemporary and multicultural foci.

**Aim:**

To develop a feasible, valid, and reliable survey tool to identify nursing students’ perceptions of being a nurse.

**Design/Methods:**

In Phase 1, a literature review and Nominal Group Technique meetings were used to generate primary survey items. Phase 2 included a pre-pilot and online pilot testing of the Perceptions of being a Registered Nurse (PRN) survey tool with 797 nursing students across all year levels at three Australian Universities.

**Results:**

The 34-item PRN survey tool uses a five-point Likert scale to measure nursing students’ perceptions of nursing, including factors influencing a nurse’s well-being, attributes and qualities of nurses, the role of the nurse, and nursing professionalism. The Item-Content validity index was high (> 0.78), and the inter-item correlation validity was identified by Pearson’s product-moment coefficient of *r* = .712. Internal reliability was confirmed with a Cronbach’s alpha = 0.83. Based upon the participation completion rate, the survey tool was deemed applicable and feasible. The majority of respondents believed that nurses have altruistic attributes; however, perceptions of nursing varied significantly when rating factors influencing the physical, emotional, and social well-being of a nurse. In later stages of training, respondents were more likely to agree that nursing is physically and emotionally demanding and that nurses experience social isolation due to shift work, finding it difficult to achieve a work-life balance.

**Conclusions:**

The PRN survey tool was found to be valid, reliable, and feasible. Future use and outcomes from PRN assessments may lead to changes to nursing curricula that enhance nursing students’ perceptions of nursing.

**Supplementary Information:**

The online version contains supplementary material available at 10.1186/s12912-023-01324-7.

## Introduction

Nursing students often enrol in higher education with altruistic, idealistic perceptions that nurses are kind, caring, compassionate, and empathetic [1, 2]. These perceptions are often influenced by personal health experiences, family, and media and are why many enrol in nursing programs [2, 3]. After graduating, newly qualified nurses soon discover that these perceptions are both naïve and mismatched to the actual role of the nurse due to workplace constraints such as heavy workloads, high acuity care, and complex care needs [4]. Upon graduation, nurses are expected to be skilled, knowledgeable, technologically savvy, able to think critically and problem-solve to meet the complex healthcare needs of a diverse and aging population [4–6]. These complex modern healthcare needs often restrict nurses’ time with their patients, depriving them of opportunities to perform altruistic roles that had initially enticed them into nursing [4, 7]. Beyond time spent with patients, new graduates also become aware of other issues previously not considered.

During the early phase of practice, newly qualified nurses soon discover that nursing can negatively impact their physical, emotional, and social well-being [4]. Some examples include sleep disturbance and social isolation imposed from working shift work [8], physical exhaustion from high workload demands [7], and emotional stress when caring for the sick and dying [9]. Other concerns are related to emotional and physical safety in the workplace, such as incivility among colleagues and verbal and physical abuse from patients and families [10]. These challenges help explain why many newly qualified nurses have difficulties transitioning from a nursing student to a qualified practitioner, and all contribute to a phenomenon known as transition shock [4, 10].

When not managed, transition shock leads to low confidence, high levels of burnout, anxiety, and stress attributing to high attrition rates [4, 11, 12]. Considering the World Health Organisation’s [13] estimated global nursing shortage of 7.6 million by the year 2030, it is clear that the factors contributing to transition shock must be further understood and addressed. Internationally, several studies have addressed the support required for newly qualified nurses during their first year of employment [10, 11, 13, 14]. Despite this, transition shock amongst newly qualified nurses remains prevalent. These concerns indicate the necessity for further exploration of other factors related to transition shock.

Ensuring new graduates develop an understanding of the contemporary nurse role and their professional identity during their undergraduate nursing education is a means of reducing transition shock [4]. With this in mind, we aimed to develop a feasible, valid, and reliable survey tool to identify students’ perceptions and changes to perceptions over the duration of the undergraduate nursing program. Findings from this survey can facilitate the evaluation of the strengths and weaknesses of nurse education programs to inform future nursing curricular development. It is hoped that the newly informed curricula will help nursing students to develop realistic perceptions of nursing prior to graduation. Further, the information gained will add insight into how students’ demographics, such as age, gender, culture, and previous health work, may influence perceptions. Finally, findings from students enrolling in nursing programs will provide nurse leaders with generalised data on how nurses are perceived within the community. This information will inform a focus on improving the public’s perception of the nurse’s professional identity.

## Methods

This project incorporated participatory co-design principles [15] to actively involve key stakeholders, nursing students, newly qualified nurses, and nursing academics in the designing, prototyping and evaluating of this survey tool. Incorporating these principles, the aim was to develop a valid, reliable, and feasible survey tool to explore nursing student’s perceptions of being a nurse and to identify if demographic variables of age, gender, year of study, previous health experiences, and main language at home influence these perceptions. This project was undertaken as part of a larger study exploring nursing students’ perceptions of being a nurse. The project included Phase 1 tool development stage and Phase 2, pilot testing.

## Ethical approval

Ethical approval for phase two of this project (pre-pilot and pilot testing) was obtained from Federation University Human Research Ethics Committee B20-052 with reciprocal approval from the University of Technology Sydney and James Cook University in accordance with Australian National Statement of Ethical Conduct in Human Research Guidelines [16]. Informed consent was obtained from all participants completing the survey. Participants were informed in the plain language information statement that their responses would be populated and reported anonymously to ensure confidentiality.

## Phase 1: survey tool development

### Step 1: literature review

A literature review was conducted to identify existing tools focussing on ‘perceptions of being a nurse’. Fourteen tools published between 1973 and 2011 (see appendix A) incorporating 446 items were found.

Overall, these existing tools were outdated, lacked contemporaneous language, and were often culturally specific. Further, the foci tended to extend beyond ‘perceptions’ to image, attitude, career ranking, gender, and qualities of nursing that did not adequately address perceptions associated with physical, emotional, and social wellbeing associated with nursing.

### Stage 2. review of published surveys

Three researchers reviewed the identified survey items. A survey tool generated by Safadi (17)] titled ‘Nursing students’ perceptions of nursing: a descriptive study of four cohorts’ was identified as the most relevant. However, this tool was found to have outdated language, did not encompass pertinent issues necessary for data analysis, and was specific to the Jordanian culture. Nevertheless, the tool did appear to be a useful foundation. The author was contacted and granted permission to use or modify some items if required. To avoid researcher bias and improve methodological rigor [24], a process of triangulation where the items developed in the PRN survey tool were compared and confirmed with relevant items in the following validated tools: Nursing attitude questionnaire (NAQ) [18, 19], Quality of Nursing Scale (QoN) [20, 21], and personal attributes and skills required for nursing [22].

### Stage 3. nominal group meetings

A Nominal Group Technique (NGT) was chosen to generate and develop appropriate items for the survey tool. Similar to the Delphi technique, this collaborative approach used appropriate stakeholders to generate ideas, explore opinions, and determine priorities to achieve a consensus [23]. The Delphi technique achieves the development of consensus through anonymous questionnaires as opposed to NGT, where stakeholders meet face-to-face to promote discussion and group exploration of ideas and priorities [23, 24].

The use of the NGT has many benefits. Participants are encouraged to identify and explore their own experiences and concepts, allowing the generation of various contributions [24]. Similar to the technique used in focus groups, during a NGT, the group discussion promotes synergy and spontaneity for participants to comment, explain, disagree, or agree and share their views [25]. These discussions promote voicing opinions that may not surface during individual interviews or anonymous surveys [25]. The researcher can also guide the discussion in line with the research aim and areas requiring further exploration [25].

The main researcher recruited a convenience sample of participants for the Nominal Group meetings from three regional healthcare organisations and one regional university, all located in Victoria, Australia. To ensure adequate stakeholder representation, the following groups were recruited; nursing students across all three-year levels currently enrolled in a Bachelor of nursing program (BN); Newly qualified nurses commencing work in a graduate nurse program at regional healthcare organisations; and nurse educators.

Five Nominal Group meetings were conducted between January and February 2020 to facilitate the development of appropriate items for the survey. The nominal group’s meetings occurred as follows:

1. Mixed group: two first, four second, and one third year BN nursing students from two different Universities, and four graduates with less than one year of experience as a registered nurse.
2. Nurse educators: Four academic nurse educators from a Victorian regional university in Australia, and two clinical nurse educators from regional healthcare organisations.
3. New Graduate nurses: Two groups of 15 (30 in total) newly qualified registered nurses who had just graduated from various higher education facilities and had just commenced employment at healthcare organisations.
4. Nursing students on enrolment: 89, first-year nursing students enrolled in a Bachelor of Nursing program before commencing any theoretical or clinical education.

The Nominal Group Technique was guided by a six-step process as described by Cooper, Cant (24)]:

1. An introduction to the project aim, including an explanation of the NGT process by a member of the research team.
2. Silent/individual generation of potential survey items on sticky notes.
3. A round table listing, and discussion of items formed on the sticky notes.
4. Group discussion and clarification of intention and terminology of items.
5. Ranking of items- participants were asked to select the most relevant five items from the generated list and rank them from 5 being most relevant to -1 less relevant.
6. Review and discussion regarding final listings.

Due to the large numbers associated with the groups listed in 3 & 4, the technique was adapted to facilitate discussion and generation of concepts. As for all groups, steps one and two were followed and facilitated by two research team members. Group 3 was then divided into two groups of fifteen, in separate rooms where a research team member facilitated the remaining steps. Next, two research team members conducted the NGT meeting for group 4 in a large auditorium. Participants were encouraged to contribute by verbalising personal perspectives and statements. All statement items were added to a list and projected on a large screen for all to see. This promoted synergy and participation. The statements were then individually reviewed and clarified.

After each meeting, the research team identified a priority list of item statements based on individual ranking and the frequency in which they were ranked. Rankings and frequency were then collated. A total of 89 items were established from the NGT meetings and were included for consideration in the next stage.

### Stage 4. selection and adaption of items

The main researcher (LA) performed an independent primary analysis of all items, followed by a tabletop thematic analysis with two other experienced researchers and one third-year nursing student [24]. The primary analysis and literature review was used as a reference check to guide discussion and analysis. Repetitious items and items not relevant to the research aim were removed. The consensus amongst the group identified four main themes: Impact on well-being, Attributes and qualities required for nursing, Nursing professionalism, and The role of the nurse. Items were then selected and reworded as necessary to generate final 34-item statements for the survey.

A five-point Likert scale was selected to best measure nursing students’ perceptions of being a nurse by enabling the transformation of subjective data to quantifiable, measurable data [26]. The ordinal measurement scale rating from (1) ‘strongly disagree’ to (5) ‘strongly agree’ facilitated quantitative data analysis [26–28]. The uneven five point scale provided participants with the option to choose a mid-point (neutral) response avoiding a forced choice of agree or disagree [26, 29]. Therefore, the higher the rating score, the more likely the participant agreed with the item statement. Conversely, the lower the rating score, the more likely the participant disagreed with the item statement. In this way, the researcher can ascertain participating students’ perceptions of being a nurse and use this as a reference for discussion. Further, as the results of PRN the survey tool is not reliant on the total score sum, this tool can be adopted to consider international cultural and professional differences.

An additional seven demographic questions were included to facilitate analysis and comparison of students’ gender, age, previous work experience in healthcare, year level of study, educational facility, and language spoken at home. Finally, three open-ended questions were added to allow students to comment in changes of perceptions and an invitation to participate in telephone interviews.

### Stage 5. tool review

To further develop the validity and feasibility of the questionnaire, a draft survey was circulated to five nursing academics and 30 third-year nursing students to calculate the item level Content Validity Index (I-CVI) and check for clarity, relevance, grammar, and language issues [15]. Participants were asked to rank each item for relevance 1 (not relevant) to 4 (highly relevant) and clarity, 1 (not clear) to 4 (totally clear). An open-ended text box was also provided for any additional comments. A I-CVI for all items exceeded > 0.78 [15].The review resulted in two other suggested items for consideration: ‘mental health nursing is emotionally demanding’ and ‘focus on the future’. The research team consensually decided that the first suggestion was aimed at a specialty area of nursing and the second did not apply to this study resulting in a non-inclusion of both.

### Stage 6. final review

A final review of the survey was conducted by all three researchers, where minor wording changes related to the open-ended questions were agreed upon. Finally, a statistician reviewed the survey tool ensuring that the statements were appropriately designed to achieve maximum analysis based on the project’s objectives.

## Phase 2: testing and survey tool validation

### Stage 1. pre-pilot testing

Pre-Pilot testing improved the survey quality by reviewing the testing design and user friendliness [30]. A purposeful sampling of 100 nursing students (34 first-year students, 33 second-year students, and 33 third-year students) from a possible population of 530 enrolled at one Australian university were invited to participate. While waiting for class, a non-academic university employee distributed paper-based surveys to nursing students to prevent participation coercion. To ensure anonymity and facilitate pre-post testing, participants were asked to mark their first three initials from their first and family names on the survey. Consenting participants were asked to place the completed surveys in a box provided. The response rate was *n* = 81 (81%).

### Stage 2. test-re-test reliability (pre-pilot)

One week later, consenting students who had completed the pre-pilot test, were once again given the survey to repeat to check for test-re-test reliability. Unfortunately, due to the Covid-19 pandemic, many students were not permitted on campus, reducing the matching sample size to *n* = 42 (51.8%). Test and re-test responses were tabled into an excel spreadsheet.

To test for reliability, the data was entered into IBM Statistical Package for the social sciences (SPSS) system vs. 26 [31] for Cronbach’s Alpha and paired-samples t-test.

### Stage 3. pilot testing

The survey tool’s final version (refer to Table 1 and Appendix B) was distributed online using Qualtrics [40]. A possible population of 4,050 nursing students enrolled in BN programs from three different universities located in three different Australian States between September 2020- March 2021 were invited to participate through their individual university program interface. Distribution was facilitated through contacts of the National Education, Simulation, and Safety (ESS) collaborative, where members hold leadership positions within BN programs. Eight hundred and forty-two nursing students responded to the survey. Survey responses that did not indicate consent 0.9% (*n* = 8) and responses from respondents that did not complete the entire survey were omitted from analysis 4.3% (*n* = 37). No attempt was made to impute missing data, resulting in 94%(*n* = 797) responses. The sample size exceeded the targeted sample size of 351 participants for a margin of error of 5% and confidence level of 95%, as recommended by Australian Bureau of Statistics].

Survey data downloaded from Qualtrics were analysed using IBM SPSS vs26 [31]. Descriptive and summary statistics (means standard deviations, Chi-square) were used to describe categorical data while group associations were explored using inferential statistics. Results with *p*-values < 0.05 were considered statistically significant. When results were less than the expected count of 5, assumptions were violated, and likelihood Ratio (LR) *p*-values substituted Chi-square *p*-values [32]. The internal consistency reliability was computed using Cronbach’s alpha. Pearson’s product moment correlation Co-efficient was used to examine inter-item correlation.

A Principle Component Analysis (PCA) was conducted to identify item statements that grouped in a linear pattern of correlations to form component factors, using a method as described by Pallant [33]. The sample exceeded the recommendation of at least 10 participants for each variable [33]. The suitability for PCA was confirmed as the Kaiser-Meyer-Olkin (KMO) value was 0.919, exceeding the recommended value of 0.6 [34, 35], and Bartlett’s [36] test of Sphericity reached statistical significance 0.05 or smaller, supporting the factorability of the correlation matrix [33]. An eigenvalue > 1 was applied to extract the number of factors and a Scree plot showed seven components. The inspection of the correlation matrix revealed the presence of many coefficients of *r* = .3 and above supporting factor analysis appropriateness.

**Table 1:** Perceptions of being a nurse survey tool (PRN)

Item statement	Strongly disagree	Disagree	Neither agree nor disagree	Agree	Strongly agree
Q1 Nursing is physically demanding					
Q2 Nursing is emotionally demanding					
Q3 Nurses experience social isolation due to shift work					
Q4 Nurses find it difficult to achieve work-life balance					
Q5 Nursing is emotionally rewarding					
Q6 Nurses work in unsafe environments (for example: physically, emotionally and culturally)					
Q7 Nurses support each other					
Q8 Nurses are required to work shifts including, weekends, and public holidays					
Q9 Nursing offers job security					
Q10 Nursing requires continual professional development					
Q11 Nursing is a profession					
Q12 Nurses are well paid					
Q13 Nurses have diverse career opportunities					
Q14 Nurses are leaders					
Q15 Nurses are respected by the community					
Q16 Nurses are ethical					
Q17 Nurses are adaptable					
Q18 Nurses are empathetic					
Q19 Nurses are kind and caring					
Q20 Nurses are respectful					
Q21 Nurses are good communicators					
Q22 Nurses are good listeners					
Q23 Nurses are resilient					
Q24 Nurses improve patients’ quality of care					
Q25 Nurses save lives					
Q26 Nurses have a lot of responsibility					
Q27 Nursing is unpredictable					
Q28 Nurses are health educators					
Q29 Nurses care for patients with an individualised perspective					
Q30 Nurses prioritise care					
Q31 Nurses advocate for their patients					
Q32 Nurses work collaboratively with other health professionals					
Q33 Nurses provide support and reassurance					
Q34 Nursing assessments influence patient care					

## Results

The validity and reliability of the PRN was based on responses from 797 nursing students who completed the survey. The demographics of this sample are outlined in Table 2. The majority of participants were female (89.1%, *n* = 710), young adults (43.7%, *n* = 348) aged between 18 and 22 years and the main language spoken at home was English 79.7% (*n* = 637). Overall, 55.6% (*n* = 443) of participants reported that they had not previously worked within the health sector, with 51.8%, (*n* = 412) participants studying at Federation University. Although there was good participation from all three-year levels of the BN program, the majority (48.6%, *n* = 387) were first year nursing students.

**Table 2:** Demographic data for sample N = 797

Variable	Category	*n*	(%)
Gender:	Male Female Other	81 710 6	10.2 89.1 0.8
Age range:	18–22 years 23–30 years 31–40 years > 40 years	348 178 155 115	43.7 22.4 19.5 14.4
Previously worked in healthcare:	Yes No	354 443	44.4 55.6
University of study:	Federation University of Technology Sydney James Cook University	412 45 339	51.8 5.7 42.5
Year level of study:	First Second Third	387 206 203	48.6 25.8 25.5
Language spoken at home:	English Language other than English (LOTE) South Asia East Asia Middle East African Europe	637 160 124 16 4 10 6	79.9 20.1 15.6 2.0 0.5 1.3 0.8

### Summary of participant ratings

Overall, Nursing students positively rated all items about statements within ‘Attributes and qualities of nurses’ and ‘The nurse’s role’. However, there was positive skewness towards item statements; 30 of 34 items were rated above a mean of 4.0 of 5 points. The highest rated item was item 11- ‘Nursing is a profession’ with a mean of 4.9, followed by item 26- ‘Nurses have a lot of responsibility’ (M = 4.73). The lowest rated items were item 6- ‘Nurses work in unsafe environments (for example physically, emotionally and culturally) (M = 3.35), item 4- ‘Nurses find it difficult to achieve work life balance’ (M = 3.37) and item 12- ‘Nurses are well paid’ (M = 3.41). These responses indicate areas requiring additional research and will be reported in depth in a later paper. Table 3 lists the means responses for each item statement.

**Table 3:** Summary statistics for nursing students’ response to the prototype PRN (n = 797)

Item Statement	Mean	Std. Deviation
Q1 Nursing is physically demanding	4.36	0.782
Q2 Nursing is emotionally demanding	4.56	0.693
Q3 Nurses experience social isolation due to shift work	3.53	1.002
Q4 Nurses find it difficult to achieve work-life balance	3.37	1.006
Q5 Nursing is emotionally rewarding	4.52	0.664
Q6 Nurses work in unsafe environments (for example: physically, emotionally and culturally)	3.35	1.089
Q7 Nurses support each other	4.12	0.824
Q8 Nurses are required to work shifts including, weekends, and public holidays	4.71	0.607
Q9 Nursing offers job security	4.40	0.765
Q10 Nursing requires continual professional development	4.84	0.415
Q11 Nursing is a profession	4.90	0.367
Q12 Nurses are well paid	3.41	1.091
Q13 Nurses have diverse career opportunities	4.64	0.589
Q14 Nurses are leaders	4.30	0.775
Q15 Nurses are respected by the community	4.41	0.723
Q16 Nurses are ethical	4.49	0.625
Q17 Nurses are adaptable	4.57	0.588
Q18 Nurses are empathetic	4.55	0.632
Q19 Nurses are kind and caring	4.57	0.622
Q20 Nurses are respectful	4.57	0.613
Q21 Nurses are good communicators	4.53	0.610
Q22 Nurses are good listeners	4.51	0.639
Q23 Nurses are resilient	4.55	0.601
Q24 Nurses improve patients’ quality of care	4.73	0.484
Q25 Nurses save lives	4.74	0.503
Q26 Nurses have a lot of responsibility	4.85	0.403
Q27 Nursing is unpredictable	4.33	0.899
Q28 Nurses are health educators	4.65	0.560
Q29 Nurses care for patients with an individualised perspective	4.45	0.712
Q30 Nurses prioritise care	4.64	0.559
Q31 Nurses advocate for their patients	4.63	0.604
Q32 Nurses work collaboratively with other health professionals	4.73	0.511
Q33 Nurses provide support and reassurance	4.72	0.505
Q34 Nursing assessments influence patient care	4.72	0.509

### Validity and reliability

The main objective in developing a survey is to demonstrate its validity and reliability. Validity can be established by demonstrating the degree of what it is intended to measure is measured using various statistical approaches and face validity [15]. Reliability of a scale indicates absence of random error and the ability to be replicated. This is generally measured with correlation tests, as explained below [15, 33].

### Internal consistency/reliability

Internal consistency reliability of the PRN survey tool was computed via SPPS v26 [31] using Cronbach’s alpha [15]. The PRN survey exhibited reliable properties, as evident by a Cronbach’s alpha of 0.836. This indicated how closely related a set of items is in assessing error in scales [15, 33]. Pearson’s product moment correlation Co-efficient examining inter-item correlation was high (*r =* .712, p = .00) (two tailed), indicating high correlation validity [17, 35].

### Test-retest reliability

A paired-samples t-test was conducted to evaluate differences in scores of the items from the same 41 participants’ responses one week apart during the pre-pilot stage. No statistically significant differences were found from Time 1 (M = 153.80, SD = 8.19) to Time 2 (M = 155.09, SD = 8.87), *t* (41) = -1.27, *p* = .21 (two tailed). The mean increase was − 1.29, with a 95% confidence interval ranging from − 3.3 to 0.75. Overall, there were no significant differences between student responses one week apart, indicating reliability in the survey items.

### Construct validity

Adequate construct validity was demonstrated through content validity and correlation validity measures which exceeded all expected values. In the development stages, the expertise of educators and students was used as a filtering mechanism to assure face validity and usability with good outcomes of I-CVI > 0.78 for all item statements [35].

### Factor analysis

Principal component analysis (PCA) was conducted to ascertain how the pattern of correlated items could identify perceptions of nursing. Seven components were initially identified as meeting Kaiser’s criterion where the eigenvalues were greater than 1 [33]. Upon further analysis of the Rotation Method, Oblimin and Kaiser Normalization, the scree plot turning points, pattern matrix, component Correlation Matrix, and parallel analysis, and based on expert statistical advice, only four of these components were considered significant [35]. (See Table 4). The rotation of factor loadings for components 1,2, 3 and 4 were all greater than the recommended threshold 0.3 for factor loading [37]. All four selected components contained at least four items and after further analysis were found to have close relationships and themes [33].

Component 1 was indicated as the strongest, with ten iterations relating to ‘attributes and qualities of a nurse.’ Item statements included within this component were 7, 16, 17, 18, 19, 20, 21, 22, 23 and 24.

Component 2 contained four iterations relating to ‘factors influencing a nurse’s well-being’ from being a nurse. However, this component indicated poor correlation with the other identified components. Item statements included within this component were 3, 4, 6, and 8.

Component 3 contained five iterations that related to ‘nursing professionalism.’ Item statements within this component included 9, 12, 13, 14, and 15.

Finally, component 4 contained seven iterations that related the ‘role of the nurse.’ Item statements included 29, 30, 31, 32, and 33.

**Table 4:** PCA Pattern Matrix (n = 797)

Item statements	Components			
	1. Attributes and qualities of a nurse	2. Factors influencing a nurse’s well-being (physical, emotional, and social)	3. Nursing professionalism	4. Role of the nurse
Q19 Nurses are kind and caring	0.871			
Q20 Nurses are respectful	0.869			
Q22 Nurses are good listeners	0.805			
Q18 Nurses are empathetic	0.787			
Q21 Nurses are good communicators	0.780			
Q16 Nurses are ethical	0.533			
Q24 Nurses improve patients’ quality of care	0.495			
Q7 Nurses support each other	0.439			
Q17 Nurses are adaptable	0.428			
Q23 Nurses are resilient	0.414			
Q4 Nurses find it difficult to achieve work-life balance		0.769		
Q3 Nurses experience social isolation due to shift work		0.725		
Q6 Nurses work in unsafe environments (for example: physically, emotionally and culturally)		0.593		
Q8 Nurses are required to work shifts including, weekends, and public holidays		0.559		
Q12 Nurses are well paid			0.673	
Q9 Nursing offers job security			0.640	
Q15 Nurses are respected by the community			0.587	
Q13 Nurses have diverse career opportunities			0.521	
Q14 Nurses are leaders			0.346	
Q31 Nurses advocate for their patients				− 0.710
Q32 Nurses work collaboratively with other health professionals				− 0.661
Q30 Nurses prioritise care				− 0.643
Q29 Nurses care for patients with an individualised perspective				− 0.628
Q33 Nurses provide support and reassurance				− 0.628
Q28Nurses are health educators				− 0.357
Q34 Nursing assessments influence patient care				− 0.321
Extraction Method: Principal Component Analysis Rotation Method: Oblimin with Kaiser Normalization: four components				

### Additional questions

To facilitate a deeper understanding as to the influences upon nursing students’ perceptions of being a nurse, the PRN tool provides the participants with an opportunity to respond to further questions: ‘Have your perceptions of being a nurse changed while studying nursing?’, ‘If they have, can you please indicate what contributed to your change in perceptions?’, and ‘Please describe in detail how your perceptions of being a nurse have changed’.

### Feasibility

The PRN tool was planned as a short online survey using plain language to increase participant availability, accessibility and participation [38]. However, during the pilot testing of the tool, there was an attrition rate of 5.2% (n = 45) who did not complete all items and data could not be used. In addition, a small number of participants did not provide consent 0.9% (*n* = 8) and 4.3% (*n* = 37) had only completed the demographic data section of the tool. Nevertheless, the overall uptake of voluntary participation in this survey exceeded recommended participation numbers [39].

In relation to completion time, noting that some participants (n = 37) did not complete the survey or may have left it open for return later, 48 outliers (duration > 1 h) and participants who had started but indicated no consent, 8 outliers (duration < 2 min) were removed. The median completion time was 16.5 min (range 3.4 to 22.8 min).

## Discussion

In addressing transition shock and high attrition rates in newly qualified nurses, nursing programs must be designed to ensure that new graduates understand the contemporary nurse role and the professional nurse identity before graduation [40]. Current health literature suggests that these accurate perceptions develop through exposure to meaningful experiences during undergraduate nursing programs [4, 40–42].

During undergraduate nursing programs, nursing students are exposed to various experiences such as theoretical lessons, simulation, and exposure to the clinical environment during mandated clinical placements. These experiences intend to enhance professional identity, competence and confidence [43]. When meaningful and purposeful, these experiences transform nursing students perceptions from idealistic to realistic [42]. Although all nursing programs must meet accreditation standards, program content and student experiences within nursing programs may differ. Differences can occur from individual academic teaching style, exposure to simulation, classroom sizes and clinical placement allocation. For example, the length of placement [44], support provided during placement [45] and exposure to learning opportunities [46] during clinical placement may be dependent on the clinical supervisor, and the size and acuity of the clinical environment. It is therefore difficult to ascertain what experiences specifically influence perceptions during the undergraduate nursing program.

By developing a feasible, valid and reliable survey tool, nursing academics responsible for curriculum development would be better informed about what influences and impacts nursing student’s development of perceptions of nursing. In this way, future nursing programs can ensure that students are exposed to relevant clinical and theoretical experiences [5]. Overall, the survey tool exhibited good validity and reliability properties in all tested aspects. For example, a construct validity of I-CVI in phase 1, stage 5 was > 0.78 was established and reliability was evident by an achievement of a Cronbach’s alpha of 0.836 in the pilot test for all item statements [35].

The PRN survey tool consists of 34 plain English language item statement questions and six demographic questions measuring four key factors- Attributes and qualities required for nursing; Factors influencing a nurse’s well-being; Nursing professionalism; and, The role of the nurse (refer to appendix B). These factors are all important in considering issues contributing to transition shock and satisfaction/dissatisfaction of nursing in newly qualified nurses. In particular, item statements addressing factors concerned with ‘*impact on wellbeing*’ from being a nurse has been highlighted by health literature in being significantly associated with transition shock and high attrition rates in newly qualified nurses [4]. This discovery confirms the need for further exploration related to nursing student experiences that influence the development of perceptions of nursing.

Although the PRN tool’s validity, reliability and feasibility has been demonstrated, the limitations during development must be recognised. The tool’s usability is based on identifying individual perceptions for each item of being a nurse. The ratings do not specify realistic and non-realistic perceptions and as such, the survey can be adopted for international use in relation to cultural and professional identity differences. Testing of this PRN tool was limited to English speaking nursing students enrolled into a Bachelor of Nursing program within Australia. Future international iterations require testing to ensure adequate translation into other languages and equitability to other nursing programs. Concurrent validity of the PRN would be further supported by conducting correlation testing with another validated tool. Finally, although the demographic questions at the beginning of the survey enabled identification of students’ year level, future iterations of the tool should include specific questions regarding the time frame over which their perceptions changed. The researchers recommend that the future use of this tool would include such detail.

Overall, an extensive use of the PRN at a national and international level would provide valuable information to improve future curriculum development to ensure that nursing students develop a better understanding of nursing before graduation. It is hoped that this will reduce high attrition rates in newly qualified nurses.

## Conclusion

In a survey of 797 Australian nursing students enrolled in a Bachelor of Nursing program, the PRN was found to be a valid, reliable and feasible survey tool across various measures. The use of the survey tool helps measure nursing student’s perceptions of being a nurse and facilitates a comparison of a variety of demographic variables such as age, gender, language spoken and year level. Future use and testing in non-English speaking countries will assist with further validation of the tool for international use. In addition, the PRN results will guide future qualitative research exploring specific nursing student experiences that are influential in developing perceptions of being a nurse in assisting with a better understanding of this phenomena.

## Electronic supplementary material

Below is the link to the electronic supplementary material.

Supplementary Material 1: Appendix A

Supplementary Material 2: Appendix B

## Data Availability

The datasets used and/or analysed during the current study are available from the corresponding author upon reasonable request.

## Abbreviations

BN: Bachelor of Nursing
ESS: National Education, Simulation and Safety
I-CVI: Item level Content Validity Index
NAQ: Nursing attitude questionnaire
NGT: Nominal group technique
PCA: Principle Component Analysis
QoN: Quality of Nursing scale
SPSS: Statistical Package for the Social Sciences
