# Supplementary material for: Perceptions of being a registered nurse (PRN): development and validation of a survey tool
Source: BMC Nurs. 2023 May 10;22:159. doi: 10.1186/s12912-023-01324-7 (PMC10170037; doi:10.1186/s12912-023-01324-7)
Supplement: Supplementary file 1 — Supplementary Material 1: Appendix A [file 12912_2023_1324_MOESM1_ESM.docx]

Appendix A: Identified instruments for evaluation of perceptions of being a nurse

| Instrument | Author / Year/ Setting |
| --- | --- |
| Schein’s Descriptive Index (SDI) | Berkery et al., 2014; Ireland [48] |
| Nursing attitude questionnaire (NAQ) | Bolan & Grainger, 2009; Canada [19]  Ten Hoeve et al., 2017; Netherlands[20] |
| Nursing orientation tool | Bolan & Grainger, 2009; Canada [19]  Ten Hoeve et al., 2017; Netherlands [20] |
| Appreciative inquiry (AI) | Chauke et al., 2015; South Africa [49] |
| Qualities of Nursing Scale (QoN) | Cowin, 2011; Australia [22]  Johnson, 2013; Australia [21] |
| Nursing Image Questionnaire | Cukljek et al., 2017; Croatia [50] |
| Nursing student’s attitudes towards older people | Henderson et al., 2008; Australia [51] |
| Nursing Professional Values Scale | Kaya et al., 2017; Turkey [52] |
| Attitudes and beliefs about Mental health problems: Professional and Public views | McCann et al., 2010; Australia [53] |
| Belgian professional self-image instrument for hospital nurses (BELIMAGE) | Milisen et al., 2010; Belgium [54] |
| Definition statement of the concept of nursing | Safadi, 2011; Jordan [18] |
| Career ranking survey | Stevens, 2011; Australia [55] |
| Personal attributes and skills required for nursing and midwifery | Waugh et al., 2014; UK [23] |
| Attitudes Toward the Nursing Profession (ATNP) | Zhang, 2008; China [56] |
