# Supplementary material for: Perceptions of being a registered nurse (PRN): development and validation of a survey tool
Source: BMC Nurs. 2023 May 10;22:159. doi: 10.1186/s12912-023-01324-7 (PMC10170037; doi:10.1186/s12912-023-01324-7)
Supplement: Supplementary file 2 — Supplementary Material 2: Appendix B [file 12912_2023_1324_MOESM2_ESM.docx]

**Appendix B: PRN Survey tool**

**Australian nursing students’ perceptions of being a nurse-PRN survey tool**

This survey aims to determine Australian nursing students' perception of being a nurse.

Thank-you for taking the time to consider participating in this study. Your contribution to this research is greatly appreciated.

Do you consent to this data being used for research purposes?

- Yes
- No

**Below is a group of questions about you. Please mark the appropriate details.**

What is your gender?

- Male
- Female
- Other

What is your age?

- 18-22 years
- 23-30 years
- 31-40 years
- >40 years

Have you previously worked within the health sector?

- Yes
- No

Please indicate the type of work experience

- Enrolled Nurse
- Ward Clerk
- Porter
- Patient Care Attendant
- Assistant in nursing
- Allied health assistant
- Phlebotomist
- Other ________________________________________________

In which year level are you enrolled? (If a combination of year levels, please indicate the highest year)

- First
- Second
- Third

What University are you studying at?

_____________________________________________________________

Please indicate your main language spoken at home

______________________________________________________________

**Click on a circle to rank your perceptions of being a nurse.**

Q1 Nursing is physically demanding

- Strongly disagree
- Somewhat disagree
- Neither agree nor disagree
- Somewhat agree
- Strongly agree

Q2 Nursing is emotionally demanding

- Strongly disagree
- Somewhat disagree
- Neither agree nor disagree
- Somewhat agree
- Strongly agree

Q 3 Nurses experience social isolation due to shift work

- Strongly disagree
- Somewhat disagree
- Neither agree nor disagree
- Somewhat agree
- Strongly agree

Q4 Nurses find it difficult to achieve work-life balance

- Strongly disagree
- Somewhat disagree
- Neither agree nor disagree
- Somewhat agree
- Strongly agree

Q5 Nursing is emotionally rewarding

- Strongly disagree
- Somewhat disagree
- Neither agree nor disagree
- Somewhat agree
- Strongly agree

Q6 Nurses work in unsafe environments (for example: physically, emotionally and culturally)

- Strongly disagree
- Somewhat disagree
- Neither agree nor disagree
- Somewhat agree
- Strongly agree

Q7 Nurses support each other

- Strongly disagree
- Somewhat disagree
- Neither agree nor disagree
- Somewhat agree
- Strongly agree

Q8 Nurses are required to work shifts including, weekends, and public holidays

- Strongly disagree
- Somewhat disagree
- Neither agree nor disagree
- Somewhat agree
- Strongly agree

Q9 Nursing offers job security

- Strongly disagree
- Somewhat disagree
- Neither agree nor disagree
- Somewhat agree
- Strongly agree

Q 10 Nursing requires continual professional development

- Strongly disagree
- Somewhat disagree
- Neither agree nor disagree
- Somewhat agree
- Strongly agree

Q 11 Nursing is a profession

- Strongly disagree
- Somewhat disagree
- Neither agree nor disagree
- Somewhat agree
- Strongly agree

Q 12 Nurses are well paid

- Strongly disagree
- Somewhat disagree
- Neither agree nor disagree
- Somewhat agree
- Strongly agree

Q13 Nurses have diverse career opportunities

- Strongly disagree
- Somewhat disagree
- Neither agree nor disagree
- Somewhat agree
- Strongly agree

Q14 Nurses are leaders

- Strongly disagree
- Somewhat disagree
- Neither agree nor disagree
- Somewhat agree
- Strongly agree

Q 15 Nurses are respected by the community

- Strongly disagree
- Somewhat disagree
- Neither agree nor disagree
- Somewhat agree
- Strongly agree

Q 16 Nurses are ethical

- Strongly disagree
- Somewhat disagree
- Neither agree nor disagree
- Somewhat agree
- Strongly agree

Q 17 Nurses are adaptable

- Strongly disagree
- Somewhat disagree
- Neither agree nor disagree
- Somewhat agree
- Strongly agree

Q 18 Nurses are empathetic

- Strongly disagree
- Somewhat disagree
- Neither agree nor disagree
- Somewhat agree
- Strongly agree

Q 19 Nurses are kind and caring

- Strongly disagree
- Somewhat disagree
- Neither agree nor disagree
- Somewhat agree
- Strongly agree

Q 20 Nurses are respectful

- Strongly disagree
- Somewhat disagree
- Neither agree nor disagree
- Somewhat agree
- Strongly agree

Q 21 Nurses are good communicators

- Strongly disagree
- Somewhat disagree
- Neither agree nor disagree
- Somewhat agree
- Strongly agree

Q 22 Nurses are good listeners

- Strongly disagree
- Somewhat disagree
- Neither agree nor disagree
- Somewhat agree
- Strongly agree

Q 23 Nurses are resilient

- Strongly disagree
- Somewhat disagree
- Neither agree nor disagree
- Somewhat agree
- Strongly agree

Q 24 Nurses improve patients’ quality of care

- Strongly disagree
- Somewhat disagree
- Neither agree nor disagree
- Somewhat agree
- Strongly agree

Q 25 Nurses save lives

- Strongly disagree
- Somewhat disagree
- Neither agree nor disagree
- Somewhat agree
- Strongly agree

Q 26 Nurses have a lot of responsibility

- Strongly disagree
- Somewhat disagree
- Neither agree nor disagree
- Somewhat agree
- Strongly agree

Q 27 Nursing is unpredictable

- Strongly disagree
- Somewhat disagree
- Neither agree nor disagree
- Somewhat agree
- Strongly agree

Q 28 Nurses are health educators

- Strongly disagree
- Somewhat disagree
- Neither agree nor disagree
- Somewhat agree
- Strongly agree

Q 29 Nurses care for patients with an individualised perspective

- Strongly disagree
- Somewhat disagree
- Neither agree nor disagree
- Somewhat agree
- Strongly agree

Q 30 Nurses prioritise care

- Strongly disagree
- Somewhat disagree
- Neither agree nor disagree
- Somewhat agree
- Strongly agree

Q 31Nurses advocate for their patients

- Strongly disagree
- Somewhat disagree
- Neither agree nor disagree
- Somewhat agree
- Strongly agree

Q 32 Nurses work collaboratively with other health professionals

- Strongly disagree
- Somewhat disagree
- Neither agree nor disagree
- Somewhat agree
- Strongly agree

Q 33 Nurses provide support and reassurance

- Strongly disagree
- Somewhat disagree
- Neither agree nor disagree
- Somewhat agree
- Strongly agree

Q 34 Nursing assessments influence patient care

- Strongly disagree
- Somewhat disagree
- Neither agree nor disagree
- Somewhat agree
- Strongly agree

|  |  |
| --- | --- |

Have your perceptions of being a nurse changed while studying nursing

- Yes
- No

If they have, can you please indicate what contributed to your change of perceptions

- Patient care experience(s)
- Positive role model(s)
- Negative role model(s)
- Lecturer(s)
- Theoretical course(s)
- Other ________________________________________________

Please describe in detail how your perceptions of being a nurse have changed

________________________________________________________________

________________________________________________________________

________________________________________________________________

________________________________________________________________

________________________________________________________________

Please feel free to add additional comments regarding your perceptions of being a nurse

________________________________________________________________

________________________________________________________________

________________________________________________________________

________________________________________________________________

________________________________________________________________

Please provide your email address if you would like to be contacted regarding participating in a phone interview or focus group.

________________________________________________________________

Please provide your email address if you would like to be entered into a draw to win an electronic tablet. This email will only be used to contact you if you win the draw.

________________________________________________________________

Thank you for your participation in this survey.
